# miR-6869-5p Inhibits Glioma Cell Proliferation and Invasion via Targeting PGK1

**DOI:** 10.1155/2020/9752372

**Published:** 2020-05-21

**Authors:** Fakai Wang, Huanjun Zhang, Bing Liu, Wei Liu, Zengchao Zhang

**Affiliations:** ^1^Brain Center, Sunshine Union Hospital of Shandong Province, Weifang 261000, China; ^2^Department of Anesthesiology, Weifang Maternal and Child Health Hospital, Weifang 261000, China; ^3^Department of Neurosurgery of Affiliated Hospital, Weifang Medical University, Weifang 261000, China; ^4^Department of Neurosurgery, Weifang Chinese Medicine Hospital, Weifang 261000, China

## Abstract

Accumulating studies have suggested the dysregulated microRNAs (miRNAs) play important roles in brain tumors, including glioma. miR-6869-5p has been documented to be aberrantly expressed in diverse cancers. However, the precise role of miR-6869-5p in glioma remains poorly understood. This study is aimed at evaluating its modifying effects on glioma. Significantly decreased expression of miR-6869-5p was found in glioma tissues and cells. Negative association was documented between miR-6869-5p and PGK1 in glioma cells, and PGK1 was demonstrated to be a targeted gene of this miRNA by luciferase reporter assay. miR-6869-5p regulated glioma cell proliferation and invasion via targeting PGK1. In addition, the survival analysis had suggested that low miR-6869-5p expression predicted poor prognosis of glioma patients. This study has suggested that miR-6869-5p is a useful tumor suppressor and prognostic marker in glioma.

## 1. Introduction

Glioma is a highly aggressive and lethal malignancy in the central nervous system. Its etiology remains largely unknown. It has been demonstrated that genetics, environmental carcinogens, and infectious factors contribute to the pathogenesis of glioma [1, 2]. Increasing exposure to ionizing radiation elevates the risk of glioma, while allergy and atopic disease history reduce the glioma risk [3]. The prognosis of glioma is poor because of the high invasion rate. Thereby, identifying potential biological markers is essential for exploring new therapeutic interventions for glioma.

During the past few decades, increasing evidence has supported the pivotal role of microRNAs (miRNAs) in malignant diseases, including glioma [4–6]. miRNAs are posttranscriptional regulators of targeted genes. Many functional miRNAs have been identified as disease markers for glioma due to their key effects on the tumorigenesis, angiogenesis, proliferation, apoptosis, and invasion of cancer cells [7, 8]. A number of well-established miRNAs are demonstrated to affect the clinical outcome, chemotherapy resistance, and radiotherapy resistance of glioma patients [9]. In particular, some miRNAs can influence tumor maintenance and progression by regulating cancer stem cells [10, 11]. Moreover, some extracellular vesicle-delivered miRNAs contribute to cell-to-cell communications in glioma, which will serve as effective therapeutic targets for glioma [12]. Accordingly, miRNAs are key regulators and promising targets for glioma. miR-6869-5p has been firstly identified to be a tumor suppressor in colorectal cancer by Yan et al. [13, 14]. In this study, we have found that miR-6869-5p was differentially expressed in glioma tissues. However, the role of miR-6869-5p in glioma remains largely unknown. The aim of the study is to demonstrate the role of miR-6869-5p on glioma cell growth and invasion. Assuring its targeted gene and regulatory mechanism in tumorigenesis would help to provide novel targets for glioma diagnosis and treatment.

## 2. Materials and Methods

### 2.1. Glioma Cases and Tumor Tissue Collection

Glioma tissues were obtained from 43 patients with glioma undergoing surgical resection between June 2015 and April 2017 at the Affiliated Hospital of Weifang Medical University and Sunshine Integration Hospital of Shandong.  Table 1 presents characteristics of all glioma patients. The follow-up time since the first surgical resection was up to 28 months. Brain samples (**n** = 43) and adjacent tissues (**n** = 43) were stored in liquid nitrogen and used for RNA extraction. The overall survival was defined as the interval between time of surgical resection and death or 28 months of the follow-up period. Our study was conducted under the government of the hospitals' ethics committee. All participants signed the written informed consent.

### 2.2. Cell Line Culture and Cell Transfection

U87 and U251 glioma cells were maintained in DMEM adding with 10% FBS (Gibco, USA). Lentivirus plasmids (PcDNA3.1) acquired from the cultural supernatant of 293T cells were used to transfected glioma cells. 8 *μ*g/ml polybrene reagent was applied for cell transfection. After incubation for 48 hours, glioma cells were harvested after incubation for 48 hours and used in the following tests. miRNA inhibitors, mimics, and corresponding controls (GeneChem, Shanghai, China) were used to treated glioma cells.

### 2.3. Real-Time Polymerase Chain Reaction (PCR)

Total RNAs were isolated from tissues and cells by the protocol of TRIzol reagent (Invitrogen, USA). cDNA synthesis was carried out by use of 2 *μ*g total RNA using a Takara RT Kit (Tianjin, China). PCR was conducted using a Takara SYBR Green Master Mix kit (Tianjin, China). A TaqMan miRNA real-time PCR assay kit (Thermo Fisher Scientific, USA) was used to detect miRNA expression. Primers used are as follows: phosphoglycerate kinase 1 (PGK1): F: 5′CTGTGGGGGTATTTGAATGG3′, R: 5′CTTCCAGGAGCTCCAAACTG3′; GAPDH: F: 5′GAGTCAACGGATTTGGTCGT3′, R: 5′TTGATTTTGGAGGGATCTCG3′.

### 2.4. Luciferase Reporter Assay

The wild-type (WT) and mutant (MT) binding sites of PGK1 3′UTR were subcloned into lentivirus plasmid vectors (pmir-GLO). The luciferase reporter system (Promega, WI, USA) was adopted to confirm the relationship of miR-6869-5p with the potential targeted gene PGK1. 293T cells were transfected for 48 hours. Experiments were conducted three times according to the protocol.

### 2.5. Cell Proliferation and Invasion

CCK-8 (Dojindo, Japan) was applied to estimate the proliferation of glioma cells according to the protocol, in which data were analyzed by the method of one-way ANOVA. Tumor invasion was assayed by transwell and scratch tests. 1 × 10^5^ cells per well were incubated in the upper and down transwell chambers (Corning, MA, USA), respectively. After 48 hours, cells were fixed with paraformaldehyde (4%) and then stained with crystal violet (0.1%). A scratch test was also carried out, cells of which were scanned under the microscope.

### 2.6. Statistical Analysis

The overall survival of glioma patients was assessed by the Kaplan-Meier analysis plus test of log-rank. Cox regression analysis by calculating the hazard ratio (HR) with 95% CI was performed to estimate the relative risk of mortality of glioma patients. We used STATA, GraphPad, and SPSS software programs for data analysis by use of Student's **t**-test or one-way ANOVA. A two tailed **P** < 0.05 was significant.

## 3. Results

### 3.1. miR-6869-5p Expression in Glioma

**Figure 1:**
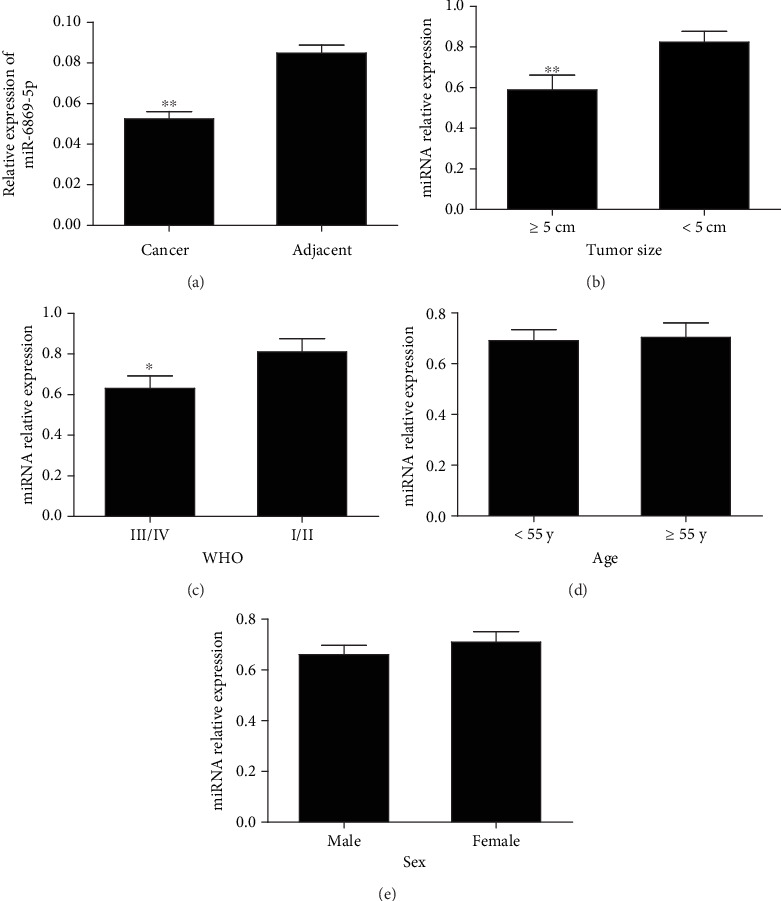
miR-6869-5p was dysregulated in glioma. (a) miR-6869-5p expression in glioma tissues was decreased in contrast to adjacent tissues (*n*/*n* = 43/43; ^∗∗^*P* < 0.01). (b) Decreased miR-6869-5p expression in glioma tissues with size ≥ 5 cm compared with tumor size < 5 cm (≥5 cm/5 cm: 18/25; ^∗∗^*P* < 0.01). (c) Decreased miR-6869-5p expression in III/IV tissues in contrast to I/II tissues (I/II: 20, III/IV: 23; ^∗^*P* < 0.05). (d) miR-6869-5p expression in glioma cases at different age (≥55/<55: 24/19). (e) miR-6869-5p expression in glioma cases of different sex (male/female: 26/17).

miR-6869-5p expression was decreased in glioma tissues (Figure 1(a)). miR-6869-5p expression was negatively related to the tumor size and WHO grade in glioma (Figures 1(b) and 1(c)), whereas no significant association was observed among patients of different age and sex (Figures 1(d) and 1(e)). Taken together, miR-6869-5p was aberrantly expressed in glioma, which might influence glioma development and progression.

### 3.2. miR-6869-5p Affected Glioma Cell Proliferation and Invasion

**Figure 2:**
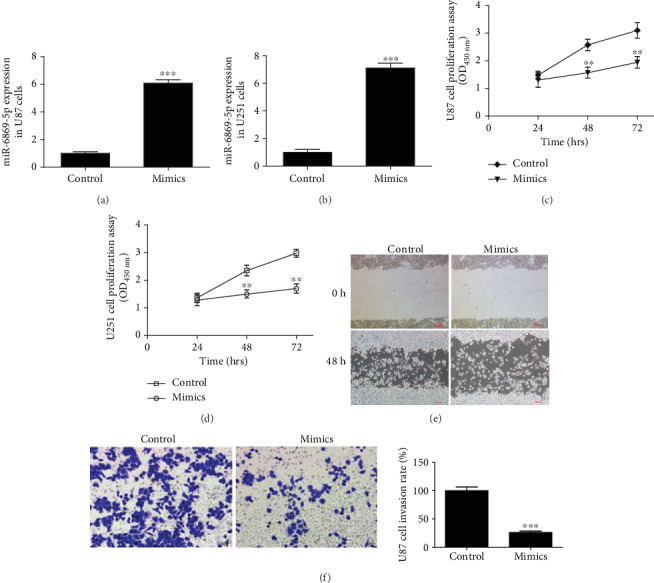
miR-6869-5p prevented the proliferation and invasion of glioma cells. (a) Real-time PCR determined miR-6869-5p expression in U87 cells (*n* = 3; ^∗∗∗^*P* < 0.001). (b) miR-6869-5p expression in U251 cells (*n* = 3; ^∗∗∗^*P* < 0.001). (c) miR-6869-5p mimics inhibited the proliferation of U87 cells (*n* = 3; ^∗∗^*P* < 0.01). (d) The proliferation of U251 cells was prevented by miR-6869-5p mimics (*n* = 3; ^∗∗^*P* < 0.01). (e) miR-6869-5p inhibited U87 glioma cells invasion (representative photos for scratch assay at 0 h and 48 h). (f) Representative pictures of transwell assay at 48 h and column analysis (*n* = 3; ^∗∗∗^*P* < 0.001).

Real-time PCR documented that miR-6869-5p expression was promoted when glioma cells were treated with miRNA mimics (Figures 2(a) and 2(b)). miR-6869-5p was capable of inhibiting glioma cell proliferation at 48 h and 72 h (Figures 2(c) and 2(d)). The invasion of mimics-treated glioma cells was inhibited compared with control cells, demonstrated by scratch assay and transwell assay (Figures 2(e) and 2(f)). All these findings suggested miR-6869-5p could prevent glioma cell proliferation and invasion.

### 3.3. PGK1 Targetedly Regulated miR-6869-5p

**Figure 3:**
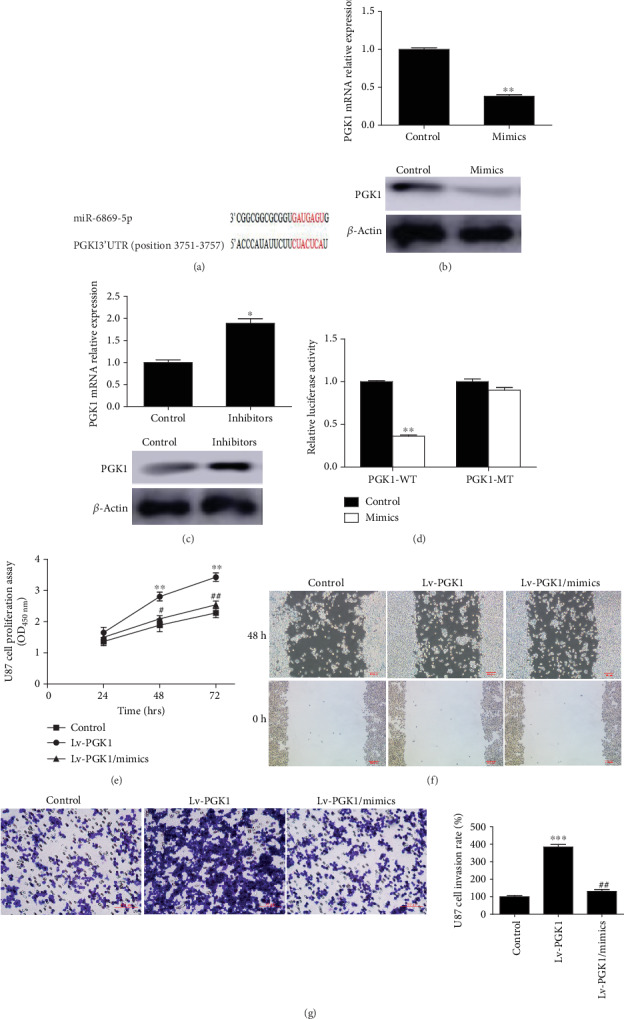
miR-6869-5p targetedly regulated PGK1 and affected cell proliferation and invasion of glioma cells. (a) Base complementary sequence between 3′UTR of PGK1 and miR-6869-5p. (b) miR-6869-5p mimics inhibited both mRNA and protein expression of PGK1 (*n* = 3; ^∗∗^*P* < 0.01; representative blotting images). (c) miR-6869-5p inhibitors promoted both mRNA and protein expression of PGK1 (*n* = 3; ^∗^*P* < 0.05; representative blotting images). (d) The luciferase reporter assay demonstrated that miR-6869-5p could regulate PGK1 expression (*n* = 3; ^∗∗^*P* < 0.01). (e) CCK-8 assay demonstrated that miR-6869-5p inhibited the proliferation of U87 glioma cells via targeting PGK1 (*n* = 3; vs. the control group, ^∗∗^*P* < 0.01; vs. the Lv-PGK1 group, ^#^*P* < 0.05; ^##^*P* < 0.01). (f) Representative figures for the scratch test at 0 h and 48 h (three independent experiments). (g) Transwell assay showed miR-6869-5p mimics inhibited U87 cell invasion by targeting PGK1 (*n* = 3; vs. the control group, ^∗∗∗^*P* < 0.001; vs. the Lv-PGK1 group, ^##^*P* < 0.01).

We performed bioinformatics analysis in the TargetScanHuman database (http://www.targetscan.org/) and found that miR-6869-5p might regulate PGK1 at the posttranscriptional level by recognizing the 3′UTR of PGK1 (Figure 3(a)). Negative association was found in glioma cells (Figures 3(b) and 3(c)). As demonstrated by the luciferase reporter assay, PGK1 could be targetedly regulated by miR-6869-5p (Figure 3(d)). In addition, miR-6869-5p could prevent glioma cell proliferation via targeting PGK1 (Figure 3(e)). Moreover, the invasion of U87 cells was significantly enhanced when PGK1 was overexpressed in cells, while miR-6869-5p mimics could rescue the effect of PGK1 by regulating its expression at the posttranscriptional level (Figures 3(f) and 3(g)). Taken together, miR-6869-5p could prevent glioma cell proliferation and invasion by targeting PGK1 in vitro.

### 3.4. Low miR-6869-5p Expression Was Associated with Poor Prognosis in Glioma

**Figure 4:**
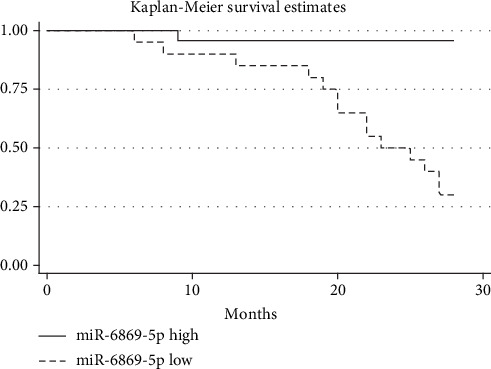
Low miR-6869-5p expression was related to poor prognosis of glioma patients (*P* < 0.001 for the log-rank test; miR-6869-5p high, the expression of miR-6869-5p is larger than 0.085 (2^-*Δ*CT^); miR-6869-5p low, the expression of miR-6869-5p is smaller than 0.053 (2^-*Δ*CT^)).

Kaplan-Meier analysis showed that glioma patients with a low level of miR-6869-5p in glioma tissues had significantly shorter overall survival (**P** < 0.001), which suggested low miR-6869-5p expression was correlated with poor prognosis in glioma (Figure 4). The Cox univariate and multivariate regression analyses suggested that glioma patients with low miR-6869-5p expression had poor prognosis (Table 2). Low miR-6869-5p expression was a risk factor for glioma independent of WHO grade and tumor size (Table 2). Accordingly, lower miR-6869-5p expression predicted poor prognosis in glioma.

## 4. Discussion

During the last decade, noncoding RNAs have been implicated in a variety of malignancies, including glioma [15–17]. miRNAs are well-known noncoding RNAs involved in tumorigenesis and cancer progression. The emerging role of miRNA in glioma has gained wide attention. Up to date, there has been no previous study investigating the effect of miR-6869-5p on glioma cells and prognosis of glioma patients. Our study has demonstrated that miR-6869-5p can regulate glioma cell proliferation and invasion by targeting PGK1. Low expression of miR-6869-5p predicts poor prognosis among glioma patients. Therefore, miR-6869-5p is a prognostic factor for glioma cases.

miRNA is a kind of single-stranded noncoding RNA with 18-25 nucleotides. Many miRNAs are differentially expressed in cancer, which participate in various cellular events by regulating the expression of targeted genes at the posttranscriptional level, such as cell growth, apoptosis, differentiation, immune surveillance, and immune escape [18–20]. Besides, a couple of miRNAs can be encapsulated and delivered by extracellular vesicles, for instance, exosomes to peripheral circulation or local immune microenvironments of cancer cells, and thus affect carcinogenesis, microenvironmental balance, and cancer progression [21, 22]. Recently, extracellular vesicles delivering miRNAs from umbilical cord mesenchymal stem cells have been identified as a potential therapy strategy due to their key effects on downregulating multiple prominent pathways associated with the glioma survival [10]. Moreover, some miRNAs regulate the fate of brain tumors by interacting with other noncoding RNAs through a competitive endogenous RNA (ceRNA) mechanism [23, 24]. In particular, networks of lncRNA-miRNA and circRNA-miRNA have been demonstrated in the carcinogenesis of glioma [24–26]. In addition, a number of miRNAs have been reported to confer modifying effects on the prognosis of glioma, such as miR-193b and miR-622 [27, 28]. As a result, miRNA plays a pivotal role in the initiation and progression of glioma. Nevertheless, the molecular mechanism by which miRNAs regulate glioma development and progression is still not fully understood. In the current study, miR-6869-5p is downregulated in glioma. It can prevent glioma cell proliferation and invasion. As evidenced by luciferase reporter assay, miR-6869-5p is capable of inhibiting glioma cell growth and invasion in vitro. Moreover, glioma patients with low miR-6869-5p expression have poor prognosis. Accordingly, miR-6869-5p is a good diagnostic and prognostic marker for glioma. However, more functional experiments and experiments in vivo are warranted to fully elucidate the effect of miR-6869-5p on the tumorigenesis and progression of glioma.

PGK1 is a glycolytic enzyme that participates in carcinogenesis, which can be produced by cancer cells and participates in regulating angiogenesis by decreasing disulfide bonds in serine protease and plasmin [29, 30]. Increased phosphorylation of PGK1 promotes tumorigenesis [29]. PGK1 is demonstrated to be a tumor promoter, which can also affect the sensitivity and resistance of cancer cells to radiotherapy and chemotherapy [31]. The study by Ding et al. shows the evidence that PGK1 enhances radioresistance in glioma cells [32]. Besides, it has been well established that PGK1 can function as a protein kinase and regulate the glycolysis metabolism of cancer cells during carcinogenesis [29, 33]. However, there are no studies investigating the role of the miRNA-PGK1 network in glioma cells. In this study, we have firstly found that PGK1 can be targetedly regulated by miR-6869-5p in glioma cells. PGK1 enhances glioma cell proliferation and invasion, while miR-6869-5p prevents glioma cell growth and invasion by downregulating PGK1. Nonetheless, we did not investigate the effect of miR-6869-5p/PGK1 on cancer cell metabolism in glioma. Future studies are encouraged to demonstrate a novel mechanism of miR-6869-5p-inhibited tumorigenesis by regulating cancer cell metabolism and targeting PGK1 or other key enzymes.

To summarize, this study supports that miR-6869-5p is a tumor suppressor in glioma, which regulates glioma cell proliferation and invasion via targeting PGK1. Low expression of miR-6869-5p predicts the poor outcome in patients with glioma. miR-6869-5p may serve as a good biomarker for glioma.

## Figures and Tables

**Table 1:** Characteristics of glioma patients.

Factors	Cases
Age (years)	
<55	19
≥55	24
Sex	
Female	17
Male	26
WHO grade	
I/II	20
III/IV	23
Tumor size (cm)	
<5	25
≥5	18

**Table 2:** Cox regression analysis of different clinicopathological variables and miR-6869-5p expression.

Factors	Univariate analysis		Multivariate analysis	
	HR (95% CI)	**P** value	HR (95% CI)	**P** value
Age (≥55 y)	1.03 (0.68-7.83)	0.462	1.15 (0.73-4.55)	0.702
Sex (male)	1.14 (0.77-4.99)	0.201	1.02 (0.52-3.62)	0.448
WHO grade (III/IV)	5.25 (0.79-8.91)	0.041	1.88 (1.01-5.06)	0.068
Tumor size (≥5)	3.23 (0.96-7.36)	0.023	2.28 (1.17-6.26)	0.045
miR-6869-5p (low)	4.47 (0.82-11.34)	0.003	1.68 (0.98-7.69)	0.020

## Data Availability

The data used to support the findings of this study are included within the article.

## References

[1] Ohgaki H., Kleihues P. (2005). Epidemiology and etiology of gliomas. *Acta Neuropathologica*.

[2] Yao L., Zhou L., Deng Y. (2019). Association between genetic polymorphisms in TYMS and glioma risk in Chinese patients: a case-control study. *OncoTargets and Therapy*.

[3] Ostrom Q. T., Bauchet L., Davis F. G. (2014). The epidemiology of glioma in adults: a "state of the science" review. *Neuro-Oncology*.

[4] Petrescu G. E. D., Sabo A. A., Torsin L. I., Calin G. A., Dragomir M. P. (2019). MicroRNA based theranostics for brain cancer: basic principles. *Journal of Experimental & Clinical Cancer Research*.

[5] Peng L., Fu J., Ming Y. (2018). The miR-200 family: multiple effects on gliomas. *Cancer Management and Research*.

[6] Yan D., Hao C., Xiao-Feng L., Yu-Chen L., Yu-Bin F., Lei Z. (2018). Molecular mechanism of notch signaling with special emphasis on microRNAs: implications for glioma. *Journal of Cellular Physiology*.

[7] Xin S., Huang K., Zhu X. G. (2019). Non-coding RNAs: regulators of glioma cell epithelial-mesenchymal transformation. *Pathology - Research and Practice*.

[8] Guo Y., Hong W., Wang X. (2019). MicroRNAs in microglia: how do microRNAs affect activation, inflammation, polarization of microglia and mediate the interaction between microglia and glioma?. *Frontiers in Molecular Neuroscience*.

[9] Rolle K. (2015). miRNA multiplayers in glioma. From bench to bedside. *Acta Biochimica Polonica*.

[10] Ngadiono E., Hardiany N. S. (2019). Advancing towards effective glioma therapy: microRNA derived from umbilical cord mesenchymal stem cells' extracellular vesicles. *Malaysian Journal of Medical Sciences*.

[11] Figueroa J., Phillips L. M., Shahar T. (2017). Exosomes from glioma-associated mesenchymal stem cells increase the tumorigenicity of glioma stem-like cells via transfer of miR-1587. *Cancer Research*.

[12] Saadatpour L., Fadaee E., Fadaei S. (2016). Glioblastoma: exosome and microRNA as novel diagnosis biomarkers. *Cancer Gene Therapy*.

[13] Yan S., Liu G., Jin C. (2018). MicroRNA-6869-5p acts as a tumor suppressor via targeting TLR4/NF-*κ*B signaling pathway in colorectal cancer. *Journal of Cellular Physiology*.

[14] Yan S., Han B., Gao S. (2017). Exosome-encapsulated microRNAs as circulating biomarkers for colorectal cancer. *Oncotarget*.

[15] Smith C. M., Catchpoole D., Hutvagner G. (2019). Non-coding RNAs in pediatric solid tumors. *Frontiers in Genetics*.

[16] Goustin A., Thepsuwan P., Kosir M., Lipovich L. (2019). The growth-arrest-specific (gas)-5 long non-coding RNA: a fascinating lncRNA widely expressed in cancers. *Noncoding RNA*.

[17] Lin Y. H. (2019). MicroRNA networks modulate oxidative stress in cancer. *International Journal of Molecular Sciences*.

[18] Cheng C. J., Bahal R., Babar I. A. (2015). MicroRNA silencing for cancer therapy targeted to the tumour microenvironment. *Nature*.

[19] Powers J. T., Tsanov K. M., Pearson D. S. (2016). Multiple mechanisms disrupt the let-7 microRNA family in neuroblastoma. *Nature*.

[20] Gorbea C., Mosbruger T., Cazalla D. (2017). A viral Sm-class RNA base-pairs with mRNAs and recruits microRNAs to inhibit apoptosis. *Nature*.

[21] Fabbri M. (2020). Natural Killer Cell–Derived Vesicular miRNAs: A New Anticancer Approach?. *Cancer Research*.

[22] Avgeris M., Panoutsopoulou K., Papadimitriou M. A., Scorilas A. (2019). Circulating exosomal miRNAs: clinical significance in human cancers. *Expert Review of Molecular Diagnostics*.

[23] Wang O., Huang Y., Wu H., Zheng B., Lin J., Jin P. (2018). LncRNA LOC728196/miR-513c axis facilitates glioma carcinogenesis by targeting tcf7. *Gene*.

[24] Yuan Y., Jiaoming L., Xiang W. (2018). Analyzing the interactions of mRNAs, miRNAs, lncRNAs and circRNAs to predict competing endogenous RNA networks in glioblastoma. *Journal of Neuro-Oncology*.

[25] Zhang R., Jin H., Lou F. (2018). The long non-coding RNA TP73-AS1 interacted with miR-142 to modulate brain glioma growth through HMGB1/RAGE pathway. *Journal of Cellular Biochemistry*.

[26] Cui B., Li B., Liu Q., Cui Y. (2017). LncRNA CCAT1 promotes glioma tumorigenesis by sponging miR-181b. *Journal of Cellular Biochemistry*.

[27] Zhu M., Zhao W., Zhao H., Zhang J. (2019). Diagnostic and prognostic value of microRNA-193b in patients with glioma and its effect on tumor progression. *Oncology Letters*.

[28] Song Q., Pang H., Qi L. (2019). Low microRNA-622 expression predicts poor prognosis and is associated with ZEB2 in glioma. *OncoTargets and Therapy*.

[29] Zhang Y., Yu G., Chu H. (2018). Macrophage-associated PGK1 phosphorylation promotes aerobic glycolysis and tumorigenesis. *Molecular Cell*.

[30] Ahmad S. S., Glatzle J., Bajaeifer K. (2013). Phosphoglycerate kinase 1 as a promoter of metastasis in colon cancer. *International Journal of Oncology*.

[31] Zhou J. W., Tang J. J., Sun W., Wang H. (2019). PGK1 facilities cisplatin chemoresistance by triggering HSP90/ERK pathway mediated DNA repair and methylation in endometrial endometrioid adenocarcinoma. *Molecular Medicine*.

[32] Ding H., Cheng Y. J., Yan H. (2014). Phosphoglycerate kinase 1 promotes radioresistance in U251 human glioma cells. *Oncology Reports*.

[33] Li X., Jiang Y., Meisenhelder J. (2016). Mitochondria-translocated PGK1 functions as a protein kinase to coordinate glycolysis and the TCA cycle in tumorigenesis. *Molecular Cell*.

